# Perceptions of antimicrobial stewardship: identifying drivers and barriers across various professions in Canada utilizing a one health approach

**DOI:** 10.3389/fpubh.2023.1222149

**Published:** 2023-08-03

**Authors:** Kayley D. McCubbin, Ellen de Jong, Anne-Marieke C. Smid, Jennifer A. Ida, Julia Bodaneze, R. Michele Anholt, Samantha Larose, Simon J. G. Otto, Herman W. Barkema

**Affiliations:** ^1^Faculty of Veterinary Medicine, University of Calgary, Calgary, AB, Canada; ^2^One Health at UCalgary, University of Calgary, Calgary, AB, Canada; ^3^College of Veterinary Medicine, Cornell University, Ithaca, NY, United States; ^4^HEAT-AMR Research Group, School of Public Health, University of Alberta, Edmonton, AB, Canada; ^5^School of Public Health, University of Alberta, Edmonton, AB, Canada

**Keywords:** antimicrobial stewardship, antimicrobial resistance, one health, Canada, drivers and barriers

## Abstract

**Introduction:**

As antimicrobial resistance (AMR) represents a substantial threat to the efficacy of available antimicrobial options, it is important to understand how to implement effective and practical mitigation efforts, including antimicrobial stewardship (AMS), across human, animal, and environmental sectors.

**Methods:**

A mixed-methods questionnaire was distributed virtually to attendees of the virtual One Health Antimicrobial Stewardship Conference (March 10–12, 2021) and their professional networks. Respondents (*n* = 81) were largely from the veterinary (75%) or human (19%) health sectors. Qualitative data were analyzed in NVivo using template analysis whereas quantitative data were analyzed in STATA using Kruskall-Wallis tests. The questionnaire asked respondents about their perceptions of AMS, as well as the perceived barriers and drivers of AMS efforts.

**Results:**

Perceptions of what AMS meant to the respondents personally and their profession as a whole were grouped into 3 main themes: 1) AMS strategies or considerations in antimicrobial prescribing and use; 2) responsibility to maintain health and preserve antimicrobial effectiveness; and 3) reducing antimicrobial use (AMU) as a goal of AMS efforts. Identified AMS barriers had 3 main themes: 1) lack of various prescribing and AMU support mechanisms; 2) shift in prescriber attitudes to drive change; and 3) stronger economic considerations to support shifting prescribing practices. Drivers of AMS had the following themes: 1) leadership to guide change; 2) education to support optimizing AMU; and 3) research to identify best practices and opportunities for action. Across all questions, 2 cross-cutting themes emerged: 1) a One Health understanding of AMS; and 2) blame placed on others for a lack of AMS success.

**Conclusion:**

Overall, sector-specific, but particularly cross-sectoral AMS drivers and barriers were identified, highlighting the importance of a One Health approach in AMR research and mitigation.

## Introduction

Human, animal, and agricultural crop health rely heavily on effective antimicrobials to treat and prevent microbial infections (1). However, pathogen resistance to antimicrobials threatens the ability to effectively treat infections in humans and animals, where global health and socio-economic impacts are projected to be substantial (1). Antimicrobial use (AMU) is the most important driver of the global increase of antimicrobial resistant (AMR) infections (2, 3). As the same active antimicrobial ingredients are used in products destined for use in humans, animals, and the environment, antimicrobial-bacterial interactions impacting AMR development are complex and multifaceted (4, 5).

The natural environment has a large role in maintaining AMR genes and organisms (5, 6). Environmental reservoirs of AMR pathogens and genes (i.e., in soil and water) represent a source of resistant genetic elements for pathogens of potential concern (7). Therefore, a One Health approach, involving key stakeholders in human, animal, and environmental sectors is required to coordinate efforts toward AMR mitigation (1).

To mitigate AMR, responsible use of antimicrobials is essential. Antimicrobial stewardship (AMS), defined as “multifaceted approaches required to sustain the efficacy of antimicrobials and minimize the emergence of AMR,” (8) is an important priority in overall AMR mitigation efforts (5, 9). Successful AMS requires coordinated actions to preserve antimicrobial effectiveness in Canada and beyond and is an important focus of the Pan-Canadian Action Plan on AMR (9). The Pan-Canadian Action Plan recognizes that Canada must take coordinated action in a One Health approach to minimize detrimental impacts of increased resistance to antimicrobials and to preserve their effectiveness (9).

Some AMS programs have been initiated in Canada, including voluntary AMU reduction initiatives, integrated AMU and AMR surveillance programs, prescribing guidelines, resources to support prescribing in a variety of health contexts, and ongoing research to support best practices, antimicrobial alternatives, and diagnostics (9–14). However, according to the most recent Canadian Antimicrobial Resistance Surveillance System Report (14), human infections with AMR pathogens of concern have increased from 2016 to 2020, including community-acquired bloodstream infections with methicillin-resistant *Staphylococcus aureus* (MRSA). In addition, the quantity (measured by weight) of medically important antimicrobials sold for use in all animals in Canada increased by 6.5% from 2019 to 2020 (14). Although reductions of AMU in some production animal industries have been described, when considering treatments based on animal weight (population corrected unit or PCU), the mg/PCU fluctuated over the last decade (14, 15). Considering the One Health implications of AMU and AMR, improving AMS in Canada in all sectors is of utmost importance.

To safeguard antimicrobial efficacy, it is crucial to improve our understanding of how to optimize using available antimicrobials in all sectors (5). Antimicrobial prescribing and use in the human and veterinary sectors are influenced by a multitude of factors including knowledge, previous experiences, and patient/client expectations, as well as broader context and norms of AMU (16–20). Broader influences include, but are not limited to, access to healthcare services, geographical area, socio-economic factors, and time constraints (19, 20). By better understanding various stakeholder perspectives of AMS in general and of ongoing AMS efforts, current initiatives could be adapted, or new initiatives developed that meet identified practical needs and improve uptake and/or impacts.

Specifically, to improve AMS efforts, it is crucial to better understand what drivers and barriers of AMS practices exist across various stakeholders to identify areas for improvement, and to guide AMS conversations and future research questions. There may also be opportunities to harmonize ongoing efforts across sectors and identify current support for a shared goal.

In March 2021, the Alberta Veterinary Medical Association, with support from Alberta Agriculture and Forestry, the AMR – One Health Consortium, and the National Collaborating Centre for Infectious Diseases, hosted a virtual One Health AMS Conference. The virtual environment facilitated a diverse complement of Canadian participants working in the human-animal-environment AMR/AMU/AMS space. To benefit from the wide variety of stakeholders groups and professions included in this event, a mixed-methods questionnaire was developed to identify: (1) perceptions of AMS from a variety of professions in Canada, and (2) drivers and barriers Canadian participants experience in their professions regarding AMS practices.

## Materials and methods

The University of Calgary Conjoint Faculties Research Ethics Board approved this study (REB21-0209).

### Participant recruitment

This study was conducted with participants of the virtual One Health Antimicrobial Stewardship Conference (March 10–12, 2021) and their professional networks. The conference included >400 attendees from 6 continents, including 26 countries, and spanned the human, animal, and environmental health sectors.

The questionnaire was advertised throughout the conference via email, and an URL and a QR code were made available through the virtual conference portal and followed an informed consent process. After the conference, a reminder email was sent to conference participants and to selected professional networks (Alberta Veterinary Medical Association and the College of Physicians and Surgeons of Alberta). The questionnaire remained open until May 15, 2021.

There were 74 of the 377 Canadian conference participants (21) that completed the questionnaire, resulting in approximately a 20% response rate, whereas 7 additional respondents did not attend the conference but were recruited through their professional networks. Only responses from Canadian participants (93%; 81/87 total participants) were included in the analyses to understand the Canadian context. Questionnaire participants were categorized into sectors (veterinary, *n* = 61; human, *n* = 15; agricultural, *n* = 2; both veterinary and human, *n* = 2, undefined = 1) based on reported profession or reported area of focus for professions that could represent any or more than one sector (i.e., academia).

### Questionnaire

A questionnaire was developed using an online survey platform (Qualtrics, Provo, UT, United States) to capture perceptions of AMS, as well as perceived drivers and barriers of AMS, as they related to the respondents’ profession. The questionnaire was developed by the One Health at UCalgary team and piloted internally.

The questionnaire contained 8 Likert scale questions where participants were asked to indicate their level of agreement regarding statements about their personal opinions of AMS, and the perceived opinion within their profession, on a 5-point scale (strongly disagree, disagree, neutral, agree, strongly agree), as well as 2 yes/no questions regarding the perceived existence of AMS drivers and barriers within their profession (see Appendix I). Not all questions were required to be completed for participants to submit their results.

To elucidate perceptions of AMS, participants were also asked the following open-ended questions: “What does antimicrobial stewardship mean to you in your profession?” and “What does antimicrobial stewardship mean to your profession as a whole?” Regarding existing barriers to AMS, participants were asked, “Do you believe there are barriers in antimicrobial stewardship in your profession?” Participants who responded ‘yes’ were asked the following: “What is preventing antimicrobial stewardship in your profession?”

To understand existing AMS drivers, participants who responded, ‘yes’ to “Do you believe there is support in place to promote/encourage antimicrobial stewardship in your profession?” were then asked, “What is currently in place that helps promote antimicrobial stewardship in your profession?”

### Data analyses

Quantitative analyses were conducted in STATA (Version 15.1, StataCorp LLC, College Station, TX, United States). In addition to inclusion of descriptive statistics, a non-parametric test (Kruskal-Wallis) was used to explore if years of experience or professional sector influenced responses to the Likert scale and to yes/no questions. Years of experience was divided into 2 categories (≤17 and > 17 years, based on the mean years of experience being 17). Statistical significance was accepted when *p* ≤ 0.05.

Qualitative data were analyzed using template analysis and a matrix analysis to elucidate differences in AMS perceptions, and AMS drivers and barriers between the sectors. Template analysis provides structure through hierarchical coding, in which sub-themes are classified under main themes (22). This approach was chosen to identify potential cross-sectoral themes and allow for sector-specific components to be coded in the hierarchical framework. Given that using a One Health approach to further understand AMS drivers and barriers in Canada is a novel approach, themes were identified through inductive coding, to enable themes to emerge organically, allowing for flexibility in coding.

In qualitative analysis, quantity of responses under each theme does not necessarily reflect their importance. Rather, the themes that emerge, regardless of how many times they emerge, is of most importance (23). Therefore, commonly mentioned AMS drivers and barriers are important, but so are those mentioned less frequently as they still impact overall AMS success.

Two researchers (KDM and JB) with NVivo (QSR International, Pty Ltd., Version 12) training conducted data coding. Preliminary inductive theme identification was done by independent review of qualitative responses and compared, followed by discussions regarding emerging themes until agreement was reached. Then, main themes and sub-themes were finalized before the first round of coding was conducted independently using NVivo by creating nodes. The second round of coding was conducted by comparing independent coding results and nodes were adapted as required. Any differences among results were discussed to ensure agreement in coding.

A matrix analysis was conducted in Microsoft Excel to organize responses to open-ended questions by sectors to evaluate potential differences in themes between sectors (24). The research team concluded that data saturation (i.e., inductive thematic saturation) was reached at data analysis completion, based on non-emergence of new themes (25).

## Results

### Participants

A total of 81 Canadians encompassing a variety of professions across the One Health spectrum participated in this study. Participants worked in the following professions/areas and could indicate >1 profession category (Table 1). Participants had a median of 15 years of experience in their profession (range: 1–45 years; mean = 17 years). A total of 74 questionnaire participants attended the virtual One Health Antimicrobial Stewardship Conference, whereas 7 did not attend.

**Table 1:** Reported professions or areas of focus of participants (participants could indicate <1), and years of experience.

Professions/areas (*N* = 81)	No.	Years of experience Mean (Range)
Veterinary clinician	36	19.9 (2–45)
Academia	31	14.7 (1–44)
Industry	12	15.6 (2–32)
Government	11	10.0 (1–25)
Medical association*	8	28.4 (12–44)
Producer	6	14.7 (2–30)
Producer association	6	17.4 (2–30)
Veterinary technician	3	10.3 (2–15)
Pharmacist	3	30.3 (25–41)
Physician	1	44
Laboratory technician	1	0

* Human or veterinary medical association.

### Quantitative data

Participants agreed that AMS was important in AMR mitigation (99%; 79/80 agreed or strongly agreed), whereas there was less agreement regarding whether their profession promotes AMS (80%; 63/79 agreed or strongly agreed), and whether it is viewed as important by their colleagues (76%; 61/80 agreed or strongly agreed) (Table 2).

**Table 2:** Participant responses to Likert scale statements regarding antimicrobial stewardship displayed on a heat map to indicate frequency of responses from least common (white) to most common (red).

Statements (*N* = 80)	Strongly disagree	Disagree	Neutral	Agree	Strongly agree
My profession is actively engaged in promoting antimicrobial stewardship^1^	1% (1)	4% (3)	15% (12)	46% (36)	34% (27)
Antimicrobial stewardship is viewed as an important consideration by my colleagues	3% (2)	5% (4)	16% (13)	52% (42)	24% (19)
I have adequate support/resources to ensure antimicrobial stewardship in my work	6% (5)	18% (14)	25% (20)	44% (35)	8% (6)
Antimicrobial stewardship is important in mitigating the threat of antimicrobial resistance	–	–	1% (1)	18% (14)	81% (65)
I believe there is more I could do personally to improve antimicrobial stewardship in my profession	1% (1)	8% (6)	26% (21)	52% (42)	13% (10)
Antimicrobial stewardship in livestock is important for human health	1% (1)	1% (1)	5% (4)	24% (19)	69% (55)
Antimicrobial stewardship in companion animals is important for human health^1^	–	1% (1)	6% (5)	32% (25)	61% (48)
Antimicrobial stewardship in humans is important for livestock health	2% (2)	2% (2)	13% (10)	35% (28)	48% (38)

1 *N* = 79.

There was agreement regarding the importance of AMS in livestock and companion animal health for maintaining human health (93%; 74/80 agreed or strongly agreed and 92%; 73/79 agreed or strongly agreed, respectively). However, agreement regarding the importance of human AMS for maintaining livestock health was slightly less (83%; 66/80 agreed or strongly agreed). Approximately half (51%; 41/80) of participants felt they had adequate support/resources to ensure AMS in their profession, and 65% (52/80) agreed there was more they could personally do to improve AMS in their profession.

Neither sector nor years of experience influenced participant responses to the majority of Likert scale statements. However, human sector participants tended to be more likely to ‘strongly agree’ whereas those in the veterinary sector were more likely to ‘agree’ (*p* = 0.06) regarding the statement that their colleagues viewed AMS as important. Veterinarians and veterinary technicians were slightly less positive (68%; 26/38 agreed or strongly agreed) compared to other professions (82%; 35/42 agreed or strongly agreed) regarding their colleagues’ views on AMS (*p* = 0.02). Further, veterinarians and veterinary technicians had a higher tendency to agree that AMS in livestock is important for human health (97%; 37/38 agreed or strongly agreed) compared to other professions (88%; 37/42 agreed or strongly agreed) (*p* = 0.052).

A total of 80% (63/79) of participants believed there were AMS barriers in their profession and 72% (56/78) believed there was AMS support in their profession. Responses by sector are in Table 3. No human sector participant responded ‘No’ regarding existence of AMS barriers and drivers. However, human sector participants with less experience (≤ 17 years) were more likely to respond “I do not know” regarding existence of AMS barriers compared to their colleagues with more experience (> 17 years) who were confident that AMS barriers existed (*p* = 0.02). There were no significant differences between sector responses to yes/no questions.

**Table 3:** Participant responses (*n* = 79) to questions on the perception of antimicrobial stewardship drivers and barriers within different professions.

	Do you believe there are barriers in improving antimicrobial stewardship in your profession?			Do you believe there is support in place to promote/encourage antimicrobial stewardship in your profession?		
Sector	Yes	I do not know	No	Yes	I do not know	No
Veterinary* (*n* = 59)	49	5	5	42	8	8
Human health (*n* = 15)	11	4	–	11	4	–
Agriculture (*n* = 2)	2	–	–	1	–	1
Human health and veterinary (*n* = 2)	1	1	–	1	1	–
Undefined (*n* = 1)	–	–	1	1	–	–

* *N* = 58 responses for “Do you believe there is support in place to promote/encourage antimicrobial stewardship in your profession?”

### Qualitative data

The matrix analysis highlighted similarities and differences across the sectors in responses. However, for questions identifying drivers and barriers, due to questionnaire design only asking participants indicating they believed either existed, reduced the number of participants responding to each open-ended question. Therefore, only participants from the veterinary and human health sectors provided responses regarding AMS drivers while one agricultural sector participant also contributed perceived AMS barriers. Therefore, qualitative responses should be considered in primarily the veterinary and human health sectors contexts, and it is indicated where differences emerged between these sectors.

### Perceptions of AMS

**Figure 1:**
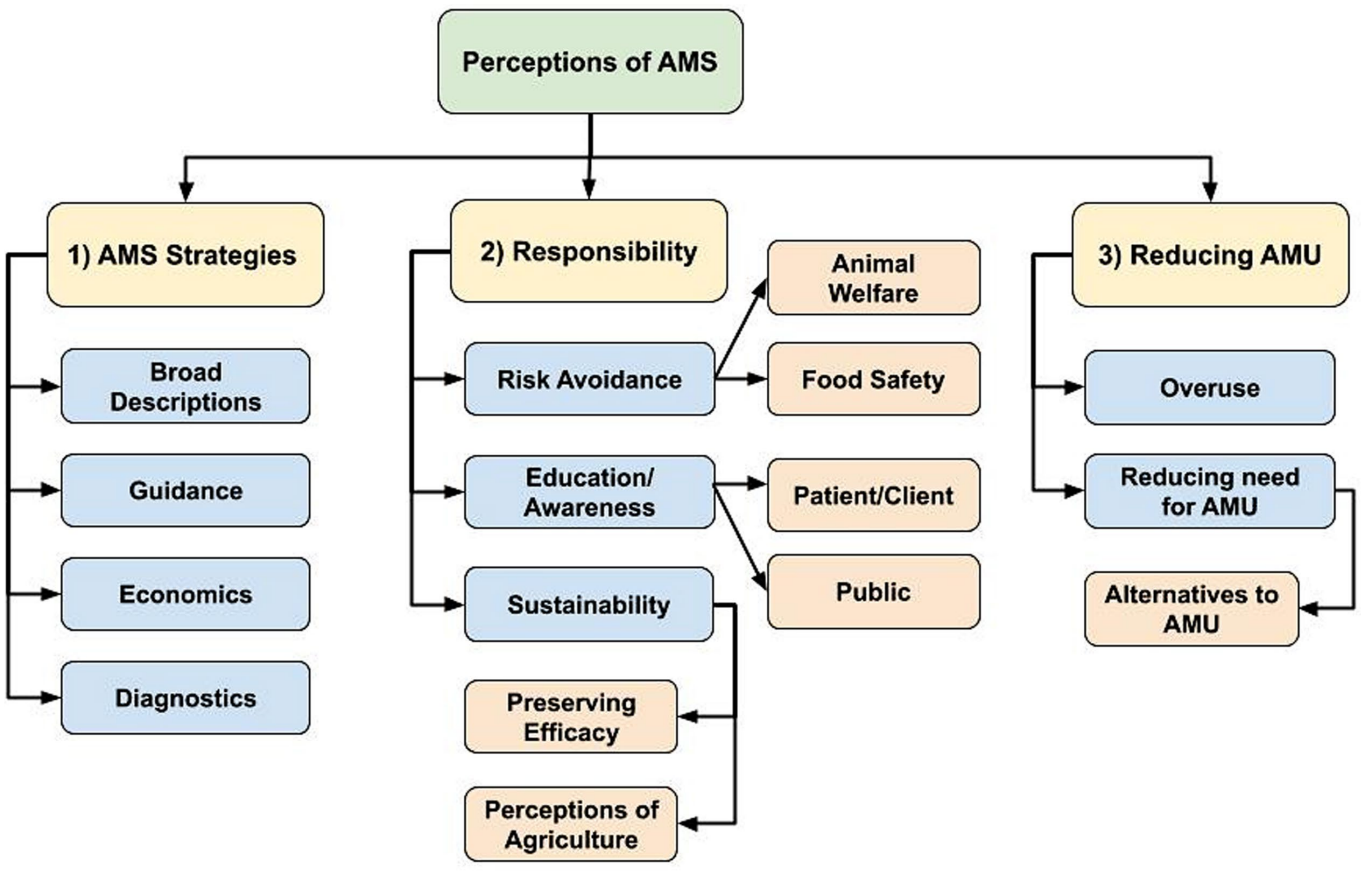
Flowchart of identified hierarchical themes across participant responses to the open-ended questions: (1) “What does antimicrobial stewardship (AMS) mean to you in your profession?” (*n* = 74; veterinary sector = 57, human health sector = 15, agricultural sector = 1, both veterinary and human health sector = 1), and (2) “What does antimicrobial stewardship mean to your profession as a whole?” (*n* = 73; one human health sector participant did not respond). (1) AMS strategies or considerations in antimicrobial prescribing and use, (2) Responsibility to maintain health and preserve antimicrobial effectiveness, (3) Reducing antimicrobial use (AMU) as a goal of AMS efforts.

Participants provided various considerations in AMS strategies and prescribing decision-making, and they shared the overarching goal of AMS as limiting AMU when possible. The following main themes emerged: (1) AMS strategies or considerations in antimicrobial prescribing and use, (2) responsibility to maintain health and preserve antimicrobial effectiveness, and (3) reducing AMU as a goal of AMS efforts. Hierarchy of themes summarizing participant responses that emerged through the inductive coding process are provided in Figure 1.

#### AMS strategies

The AMS strategies or considerations in antimicrobial prescribing and use theme represents thought processes or considerations reported as part of participant’s antimicrobial prescribing or use, or as 1 participant described: “*Striking a balance between required use and perceived need*” (Veterinary clinician, C1N41).

In their responses, participants provided broad descriptions of AMS with either concise or vague language to summarize concepts or various AMS strategies, rather than practical, actionable components (i.e., reducing inappropriate use versus increasing vaccine uptake to reduce need for AMU). Some participants included both conceptual and actionable components in their perceptions of AMS, highlighting evidence-based antimicrobial prescribing.

*“*[*Stewardship*] *Means a dynamic process of refining strategies to preserve the access to effective antimicrobials to maintain animal health and welfare. The main components of stewardship are* (1) *strategies to ensure proper use of antimicrobials when indicated. This is what we consider veterinary oversight* (*right drug, dose, duration, frequency route*)*; and* (2) *The strategies that can be implemented to avoid the use of antimicrobials when possible, such as facilities design, vaccination strategies, genetic selection, handling systems,* etc. *Stewardship initiatives also involve a large component of education and knowledge translation.”* – Veterinary clinician/Medical Association participant (C1N17).

Participants identified guiding factors influencing their AMS strategies, referring to regulations and policies aimed to reduce or control AMU, prescribing guidelines, using antimicrobials according to label instructions, and the importance of a valid patient-prescriber relationship.

Veterinary sector participants mentioned economic considerations influencing AMS strategies as they placed importance in ensuring both profitability for the producer and food affordability for the consumer.

*“Responsible and judicious use of antimicrobial products (anti-parasitic products also included) to preserve human, animal and environmental health and welfare, while ensuring the production of safe and affordable food products for human consumption.”* – Veterinary clinician (C1N59).

Diagnostics were also mentioned in both the human and veterinary sectors, primarily as the basis for antimicrobial prescribing decision-making. Specifically, bacterial culture and sensitivity testing were described as key components of AMS efforts, supporting evidence-based prescribing. Diagnostics were also mentioned regarding time limitations (i.e., the ability, or lack thereof, to provide a rapid diagnosis to guide antimicrobial choice and limited broad-spectrum AMU), and integral to ongoing AMR surveillance efforts.

#### Responsibility

It was evident in participant responses that AMS was synonymous with responsibility. This theme represented the context of AMU decisions (i.e., responsible AMU), with regards to personal or moral responsibilities participants placed on themselves regarding individual, day-to-day decisions, plus larger professional and societal duties to optimize AMU.

*“Responsible use is something the profession is focused on. Realizing we will have to use them but need to put some thought into how we are using them.” –* Industry/Human sector participant (C1N56).

The theme of risk avoidance emerged as the prescriber responsibility to maintain health of their patients via antimicrobial prescribing strategies. Food safety and animal welfare were mentioned solely by veterinary and producer participants as important considerations regarding AMU and AMS. Animal welfare was described as a moral responsibility to maintain animal health and welfare in addition to maintaining food system safety and productivity. Veterinarians and producers cited their obligations to the animals under their care, but also to humanity.

*“Protection of the public. Safeguarding and assuring availability of antimicrobials for future treatment of humans and animals.”* – Medical Association/Veterinary sector participant (C1N18).

In the human health sector, risk avoidance was also described as the prescriber’s responsibility to maintain health and increase treatment success through AMU. However, risk avoidance also referred to minimizing AMR development by encouraging or facilitating AMU.

*“In Family Medicine it means using the right antibiotic for the right patients, at the right dose for the right duration, and checking to ensure there are no harmful effects.”* – Physician/Academic/Medical Association participant (C1N40).

Education and awareness of AMS and AMR were mentioned as important personal and professional responsibilities. Specifically, antimicrobial prescribers and end-users were considered to have the responsibility to be aware of their actions and potential contributions to AMR. Further, prescribers referred specifically to continuing education (CE) being their responsibility to continually improve prescribing practices, but also that their role was to educate patients/clients and facilitate public awareness and AMS support.

*“I have a responsibility to follow guidelines regarding appropriate antimicrobial use and educate the public regarding the importance of minimizing drivers of AMR.”* – Veterinary clinician/Medical Association participant (C1N45).

*“To be [a] steward and effectively translate knowledge for public health professionals on AMR.”* – Academic/Human sector participant (C1N44).

Participants referred to AMS practices as sustainable use of antimicrobials and overall responsibility to safeguard effective treatment options for future generations. Preservation of antimicrobial efficacy was considered an integral component of AMU sustainability, as well as sustaining human and animal health in general by ensuring future access to antimicrobials.

*“The responsibility to use antimicrobials in a prudent and sustainable manner in order to preserve the use for the future and reduce current and future harm.”* – Veterinary clinician/Academic/Producer/Medical Association participant (C1N12).

Finally, the veterinary sector described their responsibility to maintain positive perceptions of agriculture, as consumer safety and animal welfare contribute to maintain a social license to use antimicrobials in animal production and to production system sustainability.

#### Reducing AMU

The goal of reducing AMU was described as both reducing antimicrobial overuse and the need for AMU. Reducing the need for antimicrobials encompassed both prevention (i.e., limiting the need for AMU through various infection prevention and health improvement initiatives) and alternative treatment options to antimicrobials.

*“Reduction of inappropriate exposure of antibiotics to help maintain antibiotic effectiveness for infection treatment.”* – Government/Human sector participant (C1N38).

*“We are looking for alternative ways to improve animal health without the use of antimicrobials.”* – Academic/Veterinary sector participant (C1N47).

Many participants viewed their role in AMS not just as ‘appropriate prescribing,’ but also as educators and facilitators promoting stewardship and preventing unnecessary AMU.

*“*To me, antimicrobial stewardship means reducing inappropriate use of antimicrobials. It means educating those who prescribe and use antimicrobials. It means questioning prescriptions when there is insufficient evidence to determine appropriateness.”* – Pharmacist/Human sector participant (C1N60).*

### Barriers to AMS

**Figure 2:**
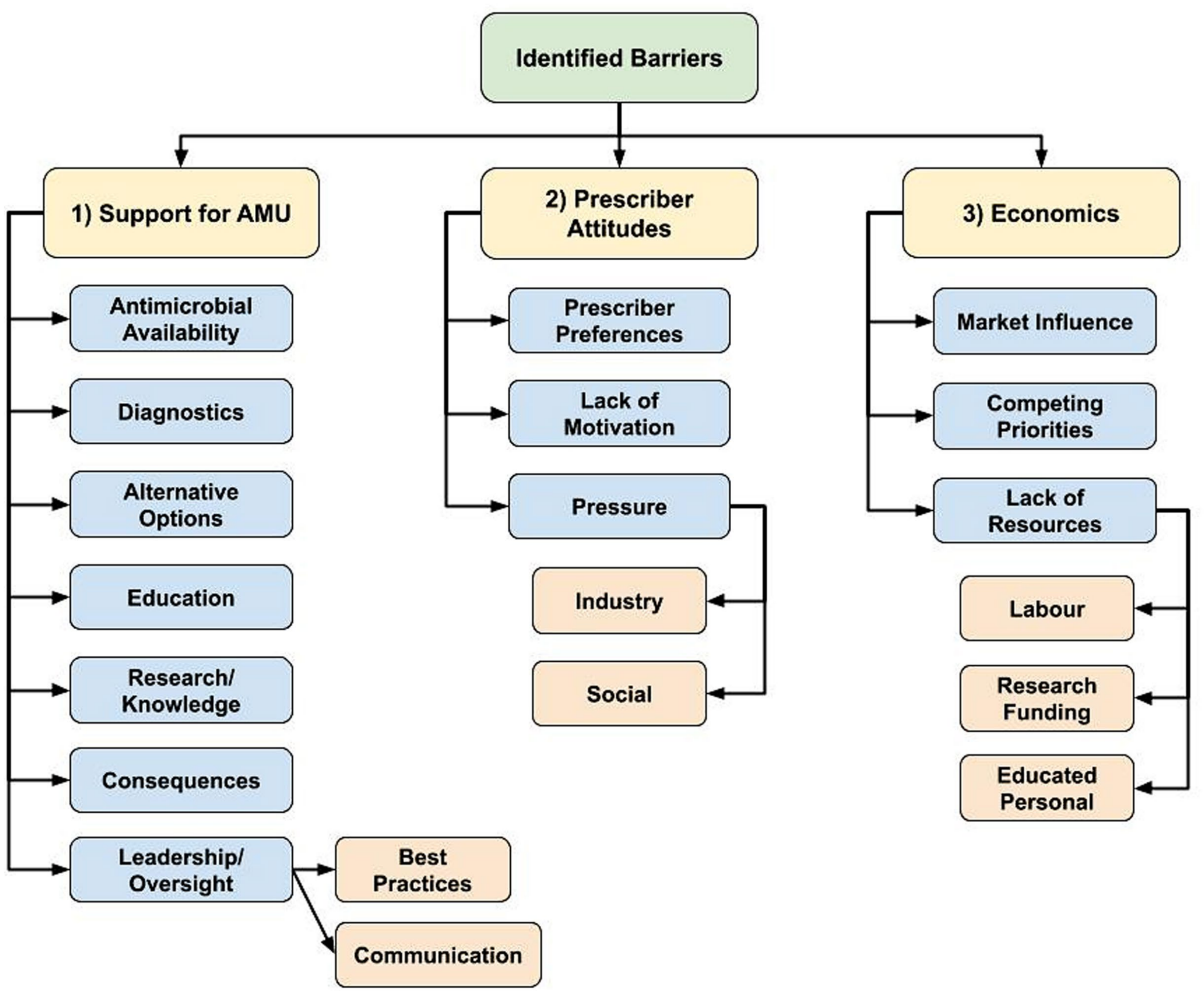
Flowchart of identified hierarchical themes across participant responses to the open-ended questions about antimicrobial stewardship barriers: “What is preventing antimicrobial stewardship in your profession?” (*n* = 59; veterinary sector = 47, human health sector = 11, agricultural sector = 1) posed to participants who responded, ‘yes’ to “Do you believe there are barriers in antimicrobial stewardship in your profession?” (1) Lack of various prescribing and antimicrobial use (AMU) support mechanisms, (2) Shift in prescriber attitudes to drive change, (3) Stronger economic considerations to support shifting prescribing practices.

Participants described a vast array of existing AMS barriers that are both sector and profession-specific but were also experienced across sectors. Regarding AMS barriers, there was emergence of 3 main themes: (1) lack of prescribing and AMU support mechanisms, (2) a required shift in prescriber attitudes to drive change, and (3) a need for stronger economic considerations to support shifting prescribing practices (Figure 2).

#### AMU support mechanisms

The AMS barrier regarding the described lack of support to optimize antimicrobial prescribing and AMU practices had various sub-themes, including the lack of access to certain antimicrobials, potentially limiting appropriate antimicrobial selection. Juxtaposition of the desire to reduce AMU coupled with required antimicrobial access for treatment was present, as well as the need for diagnostics to inform prescribing decisions. Participants expressed that widespread availability of effective antimicrobial alternatives is currently lacking.

Participants indicated they did not have access to enough educational opportunities to support their own personal knowledge as well as identified limited collective understand through limited research/knowledge in certain areas (i.e., to support development and implementation of best practices to optimize AMU and limit the need for AMU) to support required AMS education and resources.

The general lack of consequences if prescribers failed to meet AMS guidelines was identified as a barrier, or according to 1 participant, the “*intangible consequences of antimicrobial misuse*” (Pharmacist/Human sector participant C1N60). Participants described a general lack of antimicrobial prescribing oversight, and a lack of agreement regarding AMS in general, including clearly defined best practices. Participants stated if decisions were made regarding best practices, they were not communicated to enable everyone to clearly understand what is required.

*“Family practice training programs do not have strong enough emphasis and monitoring of what we do.” –* Physician/Academic/Medical Association participant (C1N40)

*“There are no simple steps or actions producers or farmers can start implementing tomorrow or this evening. As a vet tech and producer, I know I should change my farming practices, but even I don't know the first step.” –* Veterinary technician/Producer participant (C1N32)

The lack of communication and collaboration between stakeholders at various healthcare system levels was identified as a barrier, contributing to a limited shared understanding of responsibilities.

*“There is support from groups, governments, industry,* etc.*, but there is a lack of consensus and collaboration between these in their messaging and impact.” –* Veterinary clinician/Academic/Industry/Producer Organization participant (C1N35).

*“Lack of awareness and understanding between professions. It seems like at times we are ahead and at times others are. We should all be on the same page, consistently.” –* Government/Human sector participant (C1N58).

*“Engaging more stakeholders especially environmental health professionals.” –* Academic/Human sector participant (C1N44).

#### Prescriber attitudes

Prescriber attitudes and an overall lack of motivation to change behaviors were described as maintaining current levels of antimicrobial prescribing by supporting “*old habits or protocols for treatment*” (Veterinary clinical/Medical Association participant C1N45), or there being a “*lack of an overall driving force to get this done*” (Academic/Veterinary sector participant C1N28).

*“Many field practitioners may agree that antimicrobial stewardship is important but at the end of the day they do not change their behaviors due to preference, finances, external pressures,* etc.*” –* Veterinary clinician/Industry participant (C1N9).

Participants working as antimicrobial prescribers described the pressures they experience, and realities of working in healthcare. Social pressures were described as the public expectation that a healthcare visit automatically results in a prescription for them or their animal. Participants felt that a prescription has become part of the social contract of healthcare for the visit to feel like it had value. Industry pressure including intensive animal production, the pharmaceutical industry, and lack of antimicrobial alternatives were all considered to contribute to AMU.

*“Client pressure and outcome motivators put pressure on [the] profession.”* – Industry/Human sector participant (C1N56).

#### Economics

Other AMS barriers were economic in nature. This theme was primarily mentioned by the veterinary sector. Market influence, or “*economics of agricultural production*” (Veterinary clinical/Medical Association participant C1N33) was highlighted as being an important barrier, which included small profit margins and a lack of economic incentives to improve AMS.

Competing priorities were also described, such as the inherent inconsistency in private veterinary clinics between selling antimicrobials for profit and supporting AMS. Producers described being in a similarly difficult position, needing to balance fear of potential disease and profit impacts when withholding antimicrobials or limiting prophylactic AMU, and supporting AMS. One participant stated that “*Current production systems do not allow for/encourage adoption of alternative practices that may decrease/better target antimicrobial use*” (Veterinary clinician/Producer/Industry participant C1N61).

Economic limitations experienced by veterinary clients were also mentioned as limiting prescribing abilities to support AMS practices, including the cost-prohibitive nature of using diagnostic tools to optimize AMU or aid in antimicrobial selection.

*“Because of financial constraints (of clients) veterinarians often do not have culture and sensitivity results on which to base therapeutic choices, and scheduling recheck examinations can be more difficult in veterinary than in human patients.” –* Veterinary clinician/Academic participant (C1N13).

Further, the lack of cost-effective antimicrobial alternatives, and limited financial capacity to make structural changes to reduce infection rates to limit the need for antimicrobials (i.e., improvements in biosecurity or animal husbandry) were identified as important barriers. Labor constraints (i.e., time and capacity of employees) and a lack of educated personnel were also identified as reducing the ability to make improvements that support AMS.

*“A lack of cost-effective, efficient methods to address reduced use of antimicrobials is also a barrier.” –* Veterinary clinician/Government participant (C1N14).

### Drivers of AMS

**Figure 3:**
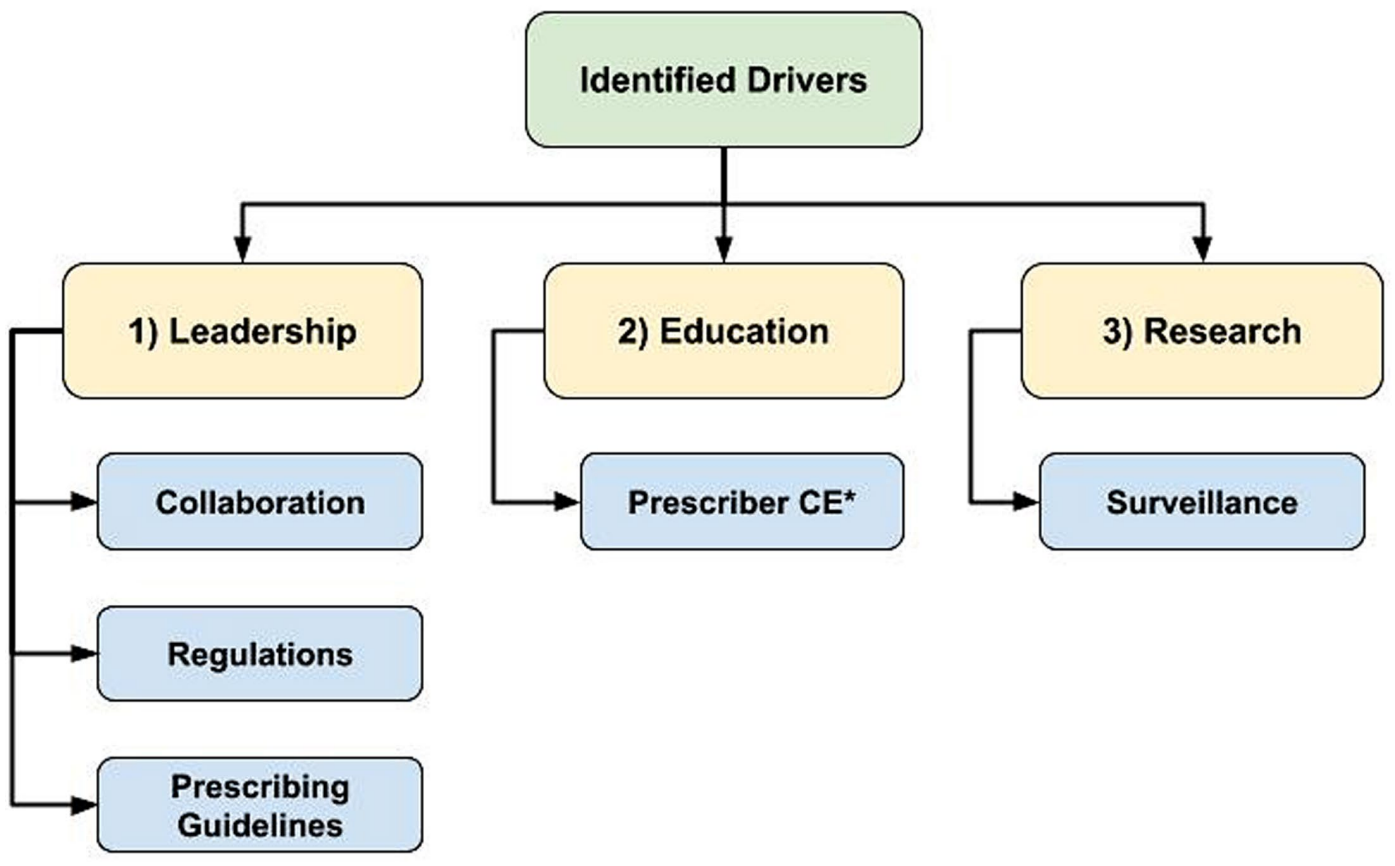
Flowchart of identified hierarchical themes across participant responses to the open-ended questions regarding antimicrobial stewardship drivers: “What is currently in place that helps promote antimicrobial stewardship in your profession?” (*n* = 47; veterinary sector = 37, human health sector = 10) asked to participants who responded ‘yes’ to “Do you believe there is support in place to promote/encourage antimicrobial stewardship in your profession?” (1) Leadership to guide change, (2) Education to support optimizing antimicrobial use, (3) Research to identify best practices and opportunities for action, *CE = Continuing Education.

Regarding drivers of AMS, there was emergence of 3 main themes: (1) leadership to guide change, (2) education to support optimizing AMU, and (3) research to identify best practices and opportunities for action (Figure 3). Whereas lack of progress in these themes presented as AMS barriers, their mention as drivers was accompanied by some examples of existing programs or support. However, the overarching theme in response to the question about existing AMS support was the general lack of support participants felt to improve AMS practices.

Examples of existing AMS leadership and guidance included regulations and professional prescribing guidelines. Additionally, examples of easily accessible educational opportunities and resources to support and drive AMS practices were provided by participants, including CE opportunities, conferences and websites. “*Guidelines and CE from professional organizations*” (Veterinary Clinician/Medical Association participant C1N10) were described as important sources of information for prescribers.

Research to better understand AMU best practices and to identify areas for AMU reduction were described as AMS drivers. Active AMU/AMR surveillance programs identifying usage trends and changes in prevalence of important resistant pathogens were also deemed important. Although participants provided some examples of existing AMS drivers, many responses indicated that there was not enough AMS support.

*“There are many programs and information available to help guide decision making, lots of CE efforts. However, the lack of specific information in some instances (ex. limited guidelines in equine practice) and lack of awareness among clinicians are remaining barriers.”–* Veterinary Clinician (C1N12). *“Written strategies exist or are being developed. More work needs to happen to promote the concepts within them.”–* Veterinary Clinician/Medical Association participant (C1N33).

### Cross-cutting themes

Across participant responses to multiple questions, there was emergence of 2 cross-cutting themes: (1) a One Health understanding of AMS, and (2) blame placed on others for the lack of AMS success. Although the transdisciplinary nature of AMR was acknowledged in responses, that also translated to blame being placed on others, including other sectors.

#### One health

Whereas questions centered around how participants perceived AMS, as well as related drivers and barriers of AMS, the One Health concept was pervasive in responses. Some descriptions of AMS included 2 sectors (primarily human and animal health), whereas others included human, animal, and environmental sectors, or specifically the term ‘One Health.’

*“Responsible and judicious use of antimicrobial products to preserve human, animal and environmental health and welfare.” –* Veterinary clinician (C1N59).

*“Practicing and educating prudent use of antimicrobials since health of all forms of life is inter-related.” – Academic/Human sector participant (C1N43).*

**Figure 4:**
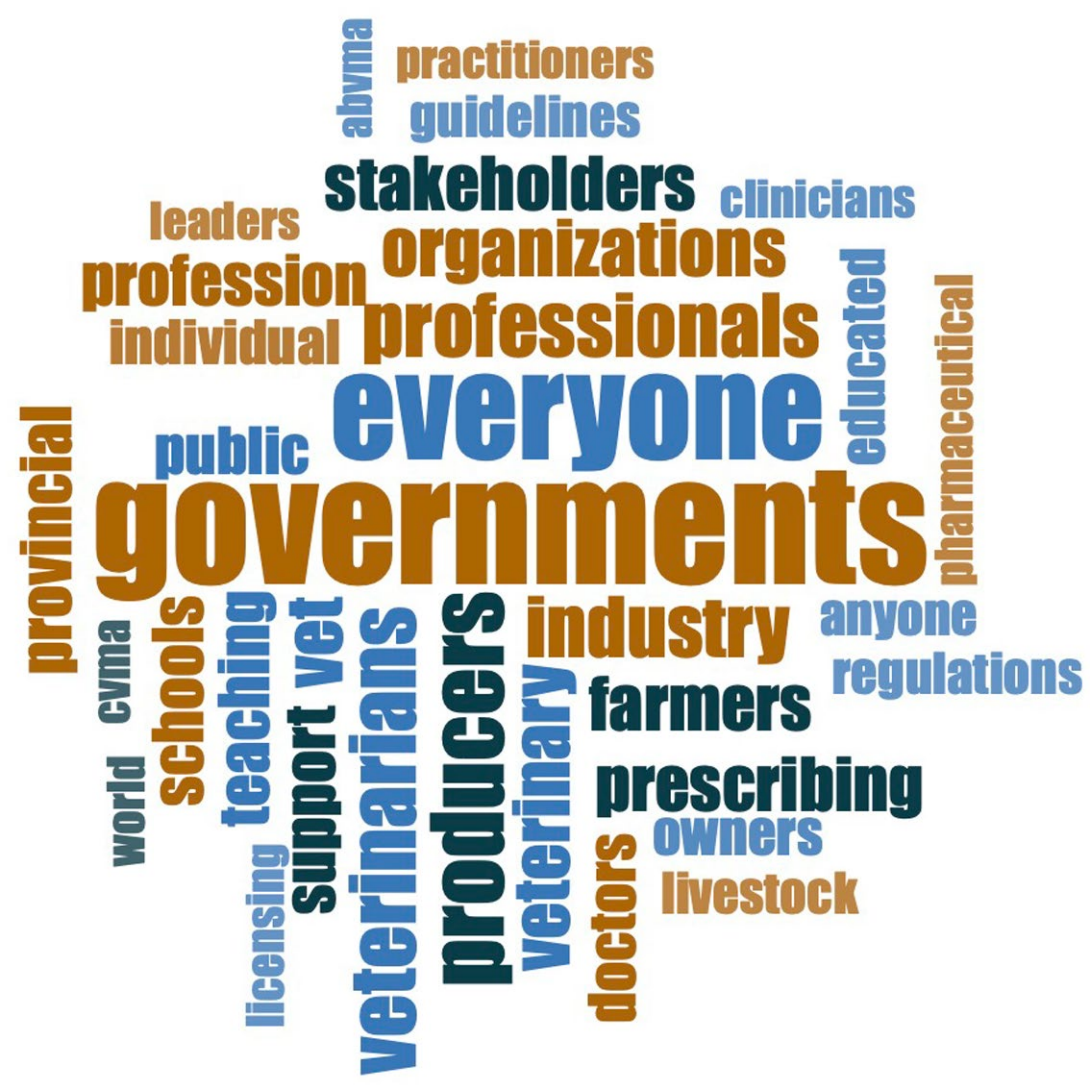
Word cloud of the most common responses (*n* = 67 participants) to the question “Who should take responsibility in promoting antimicrobial stewardship?”

The One Health theme was a pervasive response to the question “Who should take responsibility in promoting antimicrobial stewardship?” (Figure 4). Although participants indicated they believed there should be a top-down approach (i.e., government-led AMS support), they also described that everyone needs to be involved, because “*It’s One World, One Health*” (Academic/Human sector participant C1N43).

“*I think that anyone with knowledge/expertise in antimicrobial resistance should promote antimicrobial stewardship.”* – Academic/Human sector participant (C1N3)

*“Everyone has a role in antimicrobial stewardship. The lead for stewardship programs should be multidisciplinary and include health system leadership.”* – Pharmacist/Human sector participant (C1N21)

#### Blame

Another cross-cutting theme that emerged was blame. Participants placed blame for the lack of current AMS success on others within their profession, as well as on other sectors. Existing industry structures and overall cultural norms were also blamed for the lack of AMS success. Some participants (8%; 6/80) did not agree that their colleagues viewed AMS as important (Table 2), or claimed others had a “*lack of awareness and regard for the issue*” (Government/Agriculture sector participant C1N50).

*“We all have a part to play in stewardship, but not all may be putting it as a priority in the profession.” –* Academic/Veterinary sector participant (C1N46)

Additionally, blame was placed on patients and clients by prescribers for pressuring them for antimicrobial prescriptions, limiting their ability to maintain AMS practices. Prescribers also described receiving blame from patients or clients if treatments were unsuccessful.

There was also blame placed on prioritization of human health over health of other species.

*“It is not just about safeguarding certain antimicrobials for human use - need to consider impact on [the] rest of [the] species on [the] planet too.” –* Veterinary clinician/Academic/Industry/Government participant (C1N72)

Although a perceived lack of AMS support emerged as a barrier across sectors, it also emerged in response to questions regarding existing support. Participants stated that they did not have enough support in AMS activities, and that more support was required for meaningful progress.

*“More needs to be offered at the level of producers and general public.” –* Veterinary clinician (C1N22)

In addition to the perceived lack of AMS support being described in qualitative responses, it was also evident in the Likert scale responses where ~25% of participants claimed that they did not have adequate AMS support (Table 2). Participants expressed that overall, *“We have some support. But not enough.”* (Producer/Producer Organization participant C1N15).

## Discussion

This study described the presence of a ‘status quo’ of antimicrobial prescribing and use in the Canadian context, maintained by described barriers to improving AMS. Participants felt personal responsibility in AMS, but ~25% of participants did not feel they had adequate support to improve AMS. A total 80% of participants believed AMS barriers existed in their profession; the few participants indicating AMS barriers did not exist in their profession were from the veterinary sector or was a participant with an undefined profession. Human sector participants suggested that the certainty regarding the existence of AMS barriers (“I do not know” versus “Yes”) increased with time spent in the profession, which may reflect barriers individuals experience over time as they consider AMS in their profession.

Skepticism regarding AMU in animals and the subsequent impact on AMR in humans is common in the veterinary sector (26–28). However, our results indicated there was overall agreement among participants that AMS in livestock was important for humans, especially among veterinarians and veterinary technicians, but less so regarding the converse. Regardless, transmission of human AMR pathogens to animals has been identified, as well as broader impacts of human AMU and its contributions to environmental contamination and AMR are important (5, 7).

This perceived species hierarchy in AMR is reiterated in descriptions of AMS practices in livestock, where the main goal is maintaining safe food systems for humans, instead of solely focusing on animal health. In that regard, a focus on animal health to maintain human health reflects the global focus of public health where livestock AMS efforts are required to preserve antimicrobials important for human health (13, 29), but there are not necessarily policies in place to ensure the reverse. However, animal health and welfare should be prioritized, highlighted by veterinary sector participants as a moral responsibility of care and reflected in the literature (27, 28, 30).

Many participants viewed the concept of AMS to be synonymous with responsibility in terms of contributing to the AMS education of others and food safety, and most importantly, preservation of antimicrobial efficacy. However, there is an inherent contradiction in combining aims of preventing and managing bacterial infections in a risk-averse manner through antimicrobial treatment and preservation of antimicrobial efficacy for future infections (e.g., increased antimicrobial prophylaxis for COVID-19 patients during the pandemic) (31, 32). The desire to use antimicrobials to avoid potential negative clinical outcomes through practices such as prophylaxis, or ‘future discounting’ was described by UK producers and veterinarians working in a variety of livestock industries (27). Motivation to limit AMU existed but is contradicted by concern for potential animal welfare or production impacts when antimicrobials are withheld (27). Furthermore, human hospital personnel described antimicrobial prescribing being influenced by professional liability (33).

As described by participants, as a prescriber or antimicrobial user, it is difficult to assign specific negative impacts to AMU in general, or providing preventative or prophylactic antimicrobials, when impacts of increasing AMR are not immediate or clearly visible. This concern for harmful immediate impacts by withholding antimicrobials, coupled with the intangible consequences of antimicrobial misuse and the pressure put on prescribers, could contribute to an overall lack of motivation to change prescribing practices.

Unfortunately, Canadian investment in AMR has been stagnant in the past decade (34). Participants noted that it may be necessary to rethink our current health and agricultural systems to further support AMS. One important consideration is the access and cost of timely diagnostics in both the human and animal contexts, as well as the cost of other infection prevention and control measures to limit the need for antimicrobials. Further, in the current private veterinary clinic model, there is financial reliance on selling products to clients, including antimicrobials. It will be a challenge to shift our current health and agricultural systems to further support AMS from an economic perspective, although that could increase sustainability.

Specifics of how to alter each production system or healthcare context to support AMS would need to be investigated further in collaboration with stakeholders within each context. This should also include economic considerations that support sustainability of production industries as well as contribute to shared goals with pharmaceutical industries to support prolonging efficacy of antimicrobial products. As described by participants, substantial health system changes may be required to further entrench AMS priorities, including reconsidering animal production systems to improve biosecurity and reduce the need for AMU while remaining profitable, or improving market support for novel antimicrobial research and development (35).

Lack of overall leadership and stakeholder collaboration was described as an AMS barrier. Collaboration between leaders in AMS and key stakeholders at all levels in healthcare is required to effectively drive AMS efforts (36). However, prescribers’ resistance to other healthcare provider recommendations and a lack of continuity of care were identified as AMS barriers by acute care hospital personnel in Nova Scotia (33). Although a top-down approach of AMS governance was identified by participants as required for AMS improvement, they also described a need for collaboration at all levels of antimicrobial prescribers and end-users. Co-development of AMS goals and protocols within healthcare teams can serve to involve all relevant healthcare team members in the AMS discussion (33). Opportunity to influence change is a characteristic of successful implementation (37).

Increased public involvement and communication could also help limit the public pressure on prescribers for prescriptions, and limit overall antimicrobial misuse. To support efforts in AMS stakeholder communication, education in AMS efforts is integral to success; however, it should not be the sole focus of an intervention (38).

Understanding the role of the environment in the AMR ecosystem has been identified as an important knowledge gap (5). Participants identified the environmental component in AMS collaboration as lacking and that more engagement should be sought. Expanded communication and collaboration across sectors are required with a One Health approach, and essential to overall AMR mitigation success.

The cross-cutting theme of blame highlights the occasional divisions within and between sectors. Blame can contribute to feelings of apathy regarding stewardship efforts (27). ‘Other blaming’ is a common theme that emerges in AMS research, where some stakeholders feel reluctance of other stakeholders to act renders their efforts to be pointless (27, 39, 40). Antimicrobial prescribers or users could feel that their AMS efforts are being negated or diluted by the overprescribing or use of others (27). To combat feelings of apathy toward stewardship, increased transparency and accountability, or collaboration in general could help make people feel like they are working towards the same goal (27).

Finally, when asked who should take responsibility for promoting AMS, the most common response was that everyone shares responsibility in AMS efforts. The One Health concept was evident in responses, with responsibility being placed on antimicrobial prescribers and users in all sectors, as well as government, industry, professional associations, researchers, diagnosticians, and educators. Although the One Health understanding of AMR was clear and responsibility was placed on all sectors, so was blame for lack of success. However, if the barrier of poor communication and collaboration can be improved to develop a national and global sense of collective AMS responsibility, meaningful progress may be made.

The goal of the qualitative analysis was to describe responses from Canadian participants. Study design limitations included small sample sizes for the human healthcare (*n* = 15) and environmental sectors (*n* = 2) regarding quantitative responses which could have led to the overrepresentation of veterinary sector specific responses. Limitations also include potential bias for increased awareness, or belief of AMS importance and emergence of the One Health theme due to participating in an AMS-focused One Health conference (*n* = 74). Further, the virtual nature of the questionnaire limited the ability to explore participants perspectives deeper, compared to an open-ended study design conducted in person. Regardless, the study design allowed for convenient questionnaire distribution and could contribute to critical discussion of AMS barriers due to assured anonymity. Despite study limitations, results presented highlighted various themes and key components of AMS in a One Health framework to address AMR in Canada.

## Conclusion

Participants across sectors viewed AMS in Canada as important, with personal and professional responsibility and sustainability of AMU representing major themes across sectors. The described sense of responsibility can be capitalized on to prioritize AMS as “*a target to be achieved*” (Veterinary clinician/Academic participant C1N34) across sectors and professions in pursuit of a shared goal. Participants clearly identified the importance of One Health in AMS, placed blame on others and acknowledged there was more that they could do personally to improve AMS in their profession. Both sector-specific and cross-sectoral AMS drivers and barriers were identified, highlighting the diverse needs of required AMS improvements in Canada.

## Data availability statement

The datasets presented in this article are not readily available because the informed consent process referred specifically to the use of collected data for the preformed analysis and manuscript preparation. Requests to access the datasets should be directed to KM, kayley.mccubbin@ucalgary.ca.

## Ethics statement

The studies involving human participants were reviewed and approved by the University of Calgary Conjoint Faculties Research Ethics Board (REB21-0209). The patients/participants provided their written informed consent to participate in this study.

## Author contributions

KM prepared the manuscript with supervision from HB, RA, and SL. KM led the questionnaire development and study implementation, with contributions from EJ, RA, SL, SO, and HB. JB contributed to the data analysis, whereas A-MS and JI contributed significantly to the manuscript preparation. All authors contributed to manuscript revision, as well as read, and approved the submitted version.

## Funding

This work was supported by the AMR – One Health Consortium [Primarily funded by the Major Innovation Fund (MIF) program of the Alberta Ministry of Technology and Innovation].

## Conflict of interest

The authors declare that the research was conducted in the absence of any commercial or financial relationships that could be construed as a potential conflict of interest.

## Publisher’s note

All claims expressed in this article are solely those of the authors and do not necessarily represent those of their affiliated organizations, or those of the publisher, the editors and the reviewers. Any product that may be evaluated in this article, or claim that may be made by its manufacturer, is not guaranteed or endorsed by the publisher.
