# Supplementary material for: Perceptions of antimicrobial stewardship: identifying drivers and barriers across various professions in Canada utilizing a one health approach
Source: Front Public Health. 2023 Aug 3;11:1222149. doi: 10.3389/fpubh.2023.1222149 (PMC10456999; doi:10.3389/fpubh.2023.1222149)
Supplement: Supplementary file 1 [file Table_1.DOCX]

**APPENDIX I: Questionnaire**

Questionnaire was conducted in Qualtrics and followed full informed consent process.

**Demographics** *Check all that apply*

Please indicate your professional field:

| Student | MSc  PhD  DVM  MD  Nursing  Pharmacy  Other: _______ | Veterinary Medicine  Public Health  Medicine  Community Health  Animal Science  Wildlife  Other: ______________ |
| --- | --- | --- |
| Post-Doc | Veterinary Medicine  Public Health  Medicine  Community Health  Animal Science  Wildlife  Other: ______________ |  |
| Academic/researcher | Veterinary Medicine  Public Health  Medicine  Epidemiology  Microbiology  Community Health  Animal Science  Wildlife  Other: ______________ |  |
| Veterinary clinician | Practice owner  Associate  Referral hospital  Emergency  Shelter  Government  Locum  Specialty clinic  Laboratory | Companion animal  Beef cattle  Dairy cattle  Equine  Swine  Poultry  Wildlife  Aquaculture  Specialist: ___________  Other: ______________ |
| Producer | Dairy  Beef  Swine  Poultry  Aquaculture  Other: ___________ |  |
| Pharmacist |  |  |
| Producer Organization | Specify: _________ |  |
| Industry | Pharmaceuticals  Animal nutrition  Other: ____________ |  |
| Government | County | Specify: ________ |
|  | Provincial  Alberta  British Columbia  Manitoba  New Brunswick  Newfoundland  Northwest Territories  Nova Scotia  Nunavut  Ontario  Prince Edward Island  Quebec  Saskatchewan  Yukon | Specify: ______________ |
|  | National | Public Health Agency Canada  Agriculture and Agri-Food Canada  Canadian Animal Health Institute  Canadian Food Inspection Agency  Canadian Institutes of Health Research  Health Canada  NSERC  Other: ____________ |
| International | World Health Organization  Food and Agriculture Organization of the United Nations  World Organization of Animal Health  Other: __________ |  |
| Non-Governmental Organization | Country/Province: | Specify: _________ |
| Other: ___________ |  |  |

Please indicate the country in which you work: ________________

How many years of experience do you have in your indicated field? _______________________

What does ‘antimicrobial stewardship’ mean to your profession as a whole?

|  |
| --- |

What does antimicrobial stewardship mean to you in your profession?

|  |
| --- |

**Statements**

|  | **Strongly disagree** | **Disagree** | **Neutral** | **Agree** | **Strongly agree** |
| --- | --- | --- | --- | --- | --- |
| My profession is actively engaged in promoting antimicrobial stewardship. |  |  |  |  |  |
| Antimicrobial stewardship is viewed as an important consideration by my colleagues. |  |  |  |  |  |
| I have adequate support/resources to ensure antimicrobial stewardship in my work. |  |  |  |  |  |
| Antimicrobial stewardship is important in mitigating the threat of antimicrobial resistance |  |  |  |  |  |
| I believe there is more I could do personally to improve antimicrobial stewardship in my profession. |  |  |  |  |  |
| Antimicrobial stewardship in livestock is important for human health. |  |  |  |  |  |
| Antimicrobial stewardship in humans is important for livestock health. |  |  |  |  |  |

**Open ended**

Do you believe there are barriers in improving antimicrobial stewardship in your profession?

Yes

No

What is preventing antimicrobial stewardship improvement in your profession? (if yes above)

|  |
| --- |

Do you believe there is support in place to promote/encourage antimicrobial stewardship in your profession?

Yes

No

What is currently in place that helps promote antimicrobial stewardship in your profession? (if yes above)

|  |
| --- |

Who should take responsibility in promoting antimicrobial stewardship?

|  |
| --- |
